# Parameters for burst detection

**DOI:** 10.3389/fncom.2013.00193

**Published:** 2014-01-13

**Authors:** Douglas J. Bakkum, Milos Radivojevic, Urs Frey, Felix Franke, Andreas Hierlemann, Hirokazu Takahashi

**Affiliations:** ^1^Department of Biosystems Science and Engineering, ETH ZurichBasel, Switzerland; ^2^Research Center for Advanced Science and Technology, The University of TokyoTokyo, Japan; ^3^RIKEN Quantitative Biology CenterKobe, Japan; ^4^Japan Science and Technology Agency, Precursory Research for Embryonic Science and TechnologySaitama, Japan

**Keywords:** network dynamics, microelectrode array, information processing, burst detection, cell culture

## Abstract

Bursts of action potentials within neurons and throughout networks are believed to serve roles in how neurons handle and store information, both *in vivo* and *in vitro*. Accurate detection of burst occurrences and durations are therefore crucial for many studies. A number of algorithms have been proposed to do so, but a standard method has not been adopted. This is due, in part, to many algorithms requiring the adjustment of multiple *ad-hoc* parameters and further *post-hoc* criteria in order to produce satisfactory results. Here, we broadly catalog existing approaches and present a new approach requiring the selection of only a single parameter: the number of spikes *N* comprising the smallest burst to consider. A burst was identified if *N* spikes occurred in less than *T* ms, where the threshold *T* was automatically determined from observing a probability distribution of inter-spike-intervals. Performance was compared vs. different classes of detectors on data gathered from *in vitro* neuronal networks grown over microelectrode arrays. Our approach offered a number of useful features including: a simple implementation, no need for *ad-hoc* or *post-hoc* criteria, and precise assignment of burst boundary time points. Unlike existing approaches, detection was not biased toward larger bursts, allowing identification and analysis of a greater range of neuronal and network dynamics.

## Introduction

Bursts of action potentials within neurons and throughout networks are believed to serve roles in how neurons handle and store information, both *in vivo* and *in vitro*. Accurate detection of burst occurrences, durations, and boundaries are therefore crucial for many studies. *In vivo* studies have linked bursting, also referred to as “up states” in some cases, to oscillations traveling through brain regions, encoding of sensory input, assisting transmission of neural information, and markers of disease states such as epilepsy (Lisman, 1997; Izhikevich et al., 2003; Staley and Dudek, 2006). The prominent feature of primary central nervous system cells, cultured *in vitro*, is global network bursts, which are interspersed by tonic activity of varying degrees within a portion of neurons. Burst statistics have been used, for example, to study how information could become encoded after applying electrical (Madhavan et al., 2007) or chemical (Eytan et al., 2004; Selinger et al., 2004) stimulation to induce network plasticity. They have also been used to judge *in vitro* developmental stage, whereby networks transition from sparse uncorrelated spiking until displaying synchronized and aperiodic network bursts of various magnitudes in a “mature” network after 3–4 weeks (Van Pelt et al., 2004; Wagenaar et al., 2006). Both *in vivo* and *in vitro*, finite and repeating sets of activity, variously termed motifs or songs or assemblies, have been identified and proposed to be substrates to store memory traces (Baruchi and Ben-Jacob, 2004; Ikegaya et al., 2004; Segev et al., 2004; Harris, 2005; Eytan and Marom, 2006; Rolston et al., 2007; Kumar et al., 2010).

While a standard method to identify and detect network bursts has not been adopted, a number of algorithms were proposed for both *in vivo* (Legendy and Salcman, 1985; Cocatre-Zilgien and Delcomyn, 1992; Kaneoke and Vitek, 1996; Elias et al., 2007; Gourevitch and Eggermont, 2007; Ji and Wilson, 2007; Ko et al., 2012) and *in vitro* spike trains (Mukai et al., 2003; Xia et al., 2003; Segev et al., 2004; Turnbull et al., 2005; Wagenaar et al., 2005; Selinger et al., 2007; Pasquale et al., 2010; Tokdar et al., 2010; Pimashkin et al., 2011; Kapucu et al., 2012; Weihberger et al., 2013). Our working definition of a burst will be a period of high-frequency occurrences of multiple action potentials interspersed by periods of lower frequency tonic activity. We consider a *network burst* as synchronous spikes spatially distributed across multiple recording channels, including cases when few spikes occurred per channel. Burst detectors aim to build a border separating the higher and lower activity regimes. A difficulty arises because clear borders are not necessarily apparent and vary between, and even within, preparations. Many detection algorithms therefore require adjusting multiple *ad-hoc* parameters and further *post-hoc* criteria in order to produce visually acceptable results.

The various burst detection algorithms can be broadly categorized into two classes: (1) those setting a *rate-threshold* to detect bursts whenever the activity rate exceeds a specific value; (2) those setting an inter-spike-interval or *ISI-threshold* to detect bursts whenever the ISI between consecutive spikes is less than a specific value. Rate-threshold detectors simply bin together the spike times from all recording channels within a specified time window in order to create a firing rate histogram. In its most basic implementation, two parameters need to be set: the time window and the activity rate threshold. A bursting regime is then identified whenever the number of spikes exceeds the threshold (Mukai et al., 2003; Xia et al., 2003; Ji and Wilson, 2007; Pimashkin et al., 2011) or, similarly, whenever the number of active electrodes exceeds the threshold (Segev et al., 2004). This method works better for detecting multi-channel network bursts. This is due to the fact that network-wide spike trains provide higher signal-to-noise ratios than single-channel spike trains: spike counts from merged single-channel spike trains summate to multiplicatively larger values in time windows including bursts than those during non-bursting periods. The parameters are usually chosen empirically, in part, because the distribution of histogram peaks is often continuous. However, for a given time window, a rate threshold can be automatically set as the spike (or electrode) count value that separates the peaks in a bimodal probability distribution of firing rates (Ji and Wilson, 2007), also called a “discharge density” (Kaneoke and Vitek, 1996). Burst boundary time points are approximated at detection threshold crossings or via additional threshold settings. Histogram peaks and threshold crossings have been more precisely found by convolving spike times with Gaussian kernels, a decay function, or other smoothing methods (Xia et al., 2003; Segev et al., 2004; Ji and Wilson, 2007). The tendency to choose “safe” thresholds favors the detection of larger bursts, which underestimates burst number and duration.

ISI-threshold detectors consider that periods of low and high ISIs correspond to spikes occurring within and outside of bursts, respectively, and analyzing peaks in the probability distribution of the ISIs can identify appropriate thresholds (Cocatre-Zilgien and Delcomyn, 1992). The ability to assign boundary time points to specific spikes offers an advantage over rate-threshold detectors. At its most basic implementation, the ISI threshold is the only parameter required and can be automatically selected (Pasquale et al., 2010). A number of simple and complex methods to guide ISI threshold selection have been proposed. These are commonly based on finding valleys in the distributions of plain ISIs or the logarithm of ISIs or from the discharge density (Kaneoke and Vitek, 1996; Wagenaar et al., 2005; Selinger et al., 2007; Pasquale et al., 2010; Kapucu et al., 2012). The latter, actually, transforms the spike count value separating bimodal probability distributions of firing rates, as opposed to that of ISIs, to an ISI threshold by multiplying its inverse by the time window used to calculate the firing rates. Often however, a number of *post-hoc* criteria are introduced to better fit the data, including reintroducing rate-based metrics such as minimum spike counts or number of channels activated. A separate important branch of ISI threshold detectors statistically compares recorded ISIs to what would be expected assuming spike activity behaved following a model distribution. Burst regimes are then identified whenever activity exceeds expectations, termed a “surprise.” The famous Poisson Surprise method was introduced many decades ago (Legendy and Salcman, 1985) and amended variously into non-parametric Rank Surprise (Gourevitch and Eggermont, 2007), Robust Gaussian Surprise (Ko et al., 2012), and Pause Surprise (Elias et al., 2007) methods. Unlike rate-threshold detectors, ISI-threshold detectors typically operate on single-channel spike trains. Single-channel burst events detected in the first-stage are then combined together in order to identify network bursts, a process requiring additional parameters (Wagenaar et al., 2005; Pasquale et al., 2010). A main reason for the first-stage detection is because cumulative ISI distributions from multiple channels tend to average out peaks that would be apparent in probability distribution plots of single-channel spike trains. This obscures the choice of ISI threshold. Any method using a first-stage identification of single-channel bursts will also be biased toward the detection of large network bursts: network bursts composed of synchronized spikes, but with only one or a few spikes per channel, will be missed during the first-stage. Smaller “spatial” bursts of action potentials are reminiscent of the concepts of neuronal assemblies and synfire chains (Abeles et al., 1994; Kumar et al., 2010), and their detection may therefore be useful.

Our goal was to develop a simple yet robust network burst detector that does not depend on *ad-hoc* or *post-hoc* detection criteria and is able to detect smaller network bursts. Therefore, we built an *ISI*_*N*_-*threshold* detector, where *ISI*_*N*_ is the inter-spike-interval between every *N*^*th*^ spike instead of every consecutive spike. The key simple consideration is the fact that the time points of a set of *N* consecutive spikes will give a better representation of a network's firing rate status than 2 consecutive spikes (i.e., ISI). Only one detection parameter is set: the number of spikes *N* that compose the smallest network burst to consider. *N* = 10 was used throughout this paper and corresponds to approximately 0.1 spikes per recording channel for our setup (126 channels). However, ISI_*N*_-threshold detection can be performed for a wide range of *N* for both single-channel and cumulative network spike trains. Peaks in the probability distribution of ISI_*N*_ representing bursting and non-bursting regimes become more and more apparent for increasing *N*. The border between burst regimes is again automatically assigned at the valley between the (logarithmic) ISI_*N*_ peaks. Compared to an ISI threshold, an ISI_*N*_ threshold will be easier to identify, produce fewer false-positive detections, maintain precise assignment of burst boundary time points, and allow simplification of the detection algorithm. *Post-hoc* verification conditions are not required, and, like rate-threshold detectors, detection can be performed directly on the cumulative network spike train. ISI_*N*_-threshold detection is notated as: if *T*_*i* +(*N* − 1)_ – *T*_*i*_ <ISI_*N*_-threshold, then spikes *S_i_* to S_*i* +(*N* − 1)_ are in the same burst, where *T_i_* is the time of spike *S_i_*; Matlab code is provided as Supplementary Material.

We applied the ISI_*N*_-threshold detector to data gathered from cultures of rat primary neurons and glia grown over complementary metal-oxide-semiconductor (CMOS)-based microelectrode arrays (MEA), although the specific choice of recording device was not critical. From our judgment (see Discussion), the detector performed well and avoided biasing identification toward larger bursts. In many cultures, a clear distinction between large bursts across the majority of channels and smaller bursts across a subset of channels was apparent from observing distributions of the number of spikes or number of contributing channels in a burst. On the other hand, a continuum of smaller network bursts existed without a clear cutoff between bursting and non-bursting regimes. Such bursts would not be reliably identified with existing detectors, and much diversity in network information processing may be overlooked if smaller bursts are not considered. While the ISI_*N*_-threshold detector performed well by setting only a single parameter, additional criteria can improve performance. In particular, large amounts of intermittent tonic spiking will degrade the performance of most detectors, and a method to identify and exclude such channels in an optional preprocessing step is presented.

## Methods

### Cell culturing

**Figure 1:**
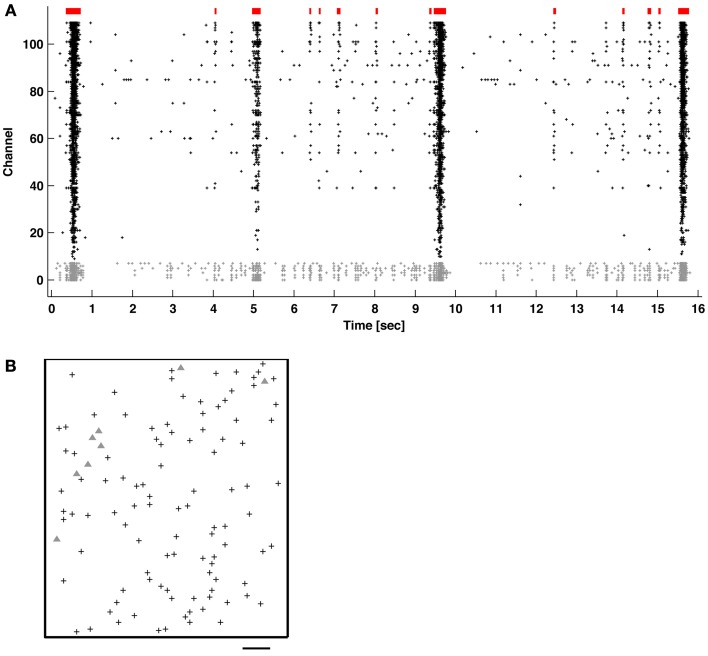
**(A)** Network bursts (indicated in red) of varying durations of coincidental APs (raster dots) recorded across multiple channels. The majority of neurons mainly fired together within network bursts (columns of dots), but some also fired “tonically” outside of bursts (channels with gray dots). **(B)** Locations of the recording electrodes selected in the CMOS-based MEA (see Methods). Gray triangles correspond to the channels with gray dots in **(A)**. Scale bar: 200 μm.

Techniques have been developed to maintain neural cultures and conduct experiments for many months (Hales et al., 2010; Bakkum et al., 2013). Briefly, E18 Wistar rat cortices were dissociated using trypsin and mechanical trituration. 20k–40k neurons and glia were seeded over an area of ~12 mm^2^ on top of the CMOS chip. Layers of poly(ethyleneimine) followed by laminin were used to adhere cells. Plating media consisted of Neurobasal-B27 supplemented with 10% horse serum and 0.5 mM GlutaMAX during the first 24 h. Growth media consisted of DMEM supplemented with 10% horse serum, 0.5 mM GlutaMAX, and 1 mM sodium pyruvate. Cultures matured for about 1 month prior to experimentation, and experiments were conducted inside an incubator to control of environmental conditions (36°C and 5% CO_2_). Burst detection was performed on 9 cultures from 4 platings. Typical network activity is depicted in Figure 1.

### CMOS-based MEA and recording of network activity

Cortical networks were grown for many weeks over 11,011-electrode CMOS-based MEAs (Frey et al., 2010; Livi et al., 2010), which provide enough spatial and temporal resolution to detect action potentials from any neuron lying on the array: 1.8 × 2.0 mm^2^ area containing 8.2 × 5.8 μm^2^ electrodes with 17.8 μm pitch (3150 electrodes per mm^2^), sampled at 20 kHz. Subsets of 126 electrodes can be read-out (and/or stimulated) at one time, and electrode selection can be re-configured within a few ms. Custom software on a personal computer, including modified Meabench code, a field-programmable gate array (FPGA), and a microcontroller embedded in a custom circuit board were used to acquire data (Wagenaar et al., 2005; Muller et al., 2013). To identify the locations of neurons growing over the array, a sequence of about one hundred recording configurations were scanned across the whole array while recording spontaneous activity. To sample network activity, 126 recording electrodes were arbitrarily selected. Because an action potential from a single soma can be detected on multiple nearby electrodes, caution is required if configured electrodes are close to each other: activity from a single soma could be falsely detected as a small network burst. This case was avoided by maintaining a minimum inter-recording-electrode distance exceeding the spatial spread of somatic signals (about 40 μm). Alternatively, this case could be avoided by transforming channel spike trains into neuronal spike trains via *spike sorting* techniques (Franke et al., 2012a,b; Jackel et al., 2012). Matlab R2012a was used for data analysis.

### Proposed network burst detection algorithm

**Figure 2:**
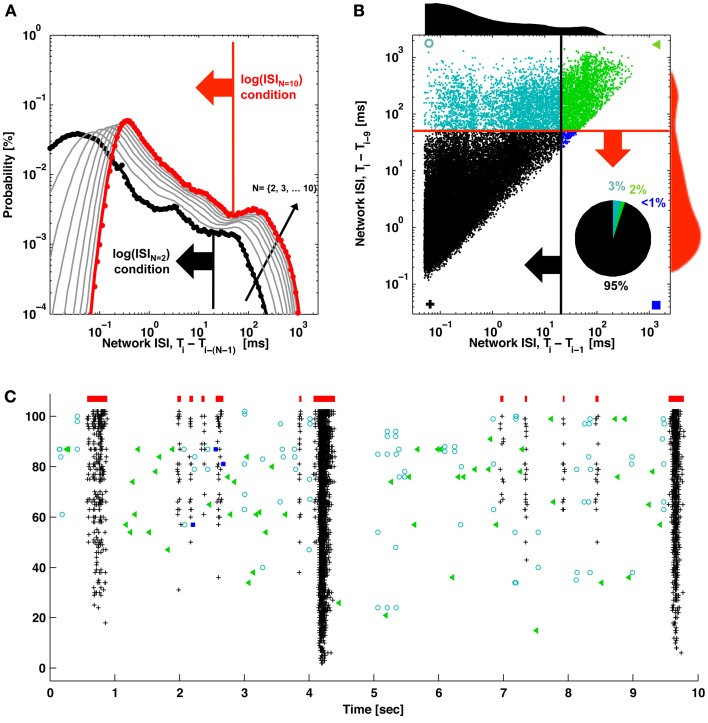
**ISI_*N*_ thresholds and burst detection. (A)** The probability of elapsed times between consecutive spikes (black; ISI) and every *N*th spike (gray) up to every 10th spike (red) are plotted. Elevated firing during network bursting corresponds to lower ISI, and the red arrow indicates the threshold for burst detection used in Figure 1. **(B)** The elapsed time between consecutive spikes is plotted vs. the elapsed time between every 10 spikes. Histograms correspond to the black and red probability distributions in **(A)**, and the red and black arrows correspond to the ISI and ISI_*N*_ thresholds in **(A)**. For **(A)** and **(B)**, ISIs were jittered by a random value between zero and one sample (50 μs) in order to better visualize the contribution from low ISIs. These would otherwise be plotted on top of each other in discrete lines corresponding to multiples of the sampling rate. The inset pie chart shows the percentage of spikes in each quadrant. Symbols match the spike markers in **(C)**. **(C)** Detector performance for a segment of network activity. Black pluses and blue squares indicate spikes that would be classified in bursts (red bars) using an ISI_*N* = 10_ threshold. Black pluses and cyan circles indicate spikes that would be classified in bursts according to an ISI_*N* = 2_ threshold. Green triangles indicate spikes outside of bursts for either case.

The proposed ISI_*N*_-threshold method to detect bursts is depicted in Figure 2, and Matlab code is provided as Supplementary Material. To summarize, bursting (low ISI_*N*_) and non-bursting regimes (high ISI_*N*_) typically formed two peaks in a histogram of the base-10 logarithm of ISI_*N*_. ISI_*N*_ is the inter-spike-interval between every *N*th spike in the network. The time points from all spikes on all channels were combined into a single train and input to the detector; spatial information about electrode locations was not incorporated. Negative signals exceeding 5 standard deviations of the noise were considered to be spikes arising from somatic action potentials. The border between regimes, or ISI_*N*_ threshold, was chosen as the valley between the peaks (or the first minima for multiple peaks). Setting histogram bin widths to be equally spaced on a logarithmic scale increases the height of the non-bursting (high ISI_*N*_) peak, providing better discrimination (Selinger et al., 2007; Pasquale et al., 2010). The user must choose a single parameter: the number of spikes *N* that compose the smallest network burst to consider. Detection is simply: If *N* consecutive spikes occur within a time period equal to or less than the ISI_*N*_ threshold, the spikes are assigned to a burst. The burst ends when this condition is no longer met. Since all spikes are assigned to be within or outside of a burst, specific time points for the first and last spikes in a burst are assigned. For this paper, we chose *N* = 10 spikes, and identified ISI_*N*_ thresholds from 1 h recordings. Channels with high levels of tonic spiking were excluded prior to analysis as depicted in Figure 5. This corresponded to between 2 and 10 percent of the recording channels (indicated by gray markers in raster plots).

### Existing burst detection algorithms used for comparison

#### Rate-threshold detector

A *rate-threshold detector* algorithm was adapted from the work of Ji and Wilson (2007). Specifically, a network burst was detected if a firing rate histogram with 50 ms or 5 ms time windows exceeded *N* spikes. Rate-thresholds, *N*, were automatically set at the valley between peaks in the spike count (or electrode count) probability distributions. A 50 ms time window corresponds to the choice by Mukai et al. (2003), who used a similar preparation to ours. A shorter time window (e.g., 5 ms) may allow more precise identification of burst start and end times.

#### ISI-threshold detector

An *ISI-threshold detector* algorithm was adapted from pseudo code provided in a recent and thorough publication by Pasquale et al. (2010), who also used a similar preparation. In the first-stage single-channel burst detection, ISI thresholds were automatically set at a valley between peaks in the (logarithmic) ISI probability distribution and capped at maximum value of 100 ms. A single-channel burst was detected if the ISIs of 5 consecutive spikes were each less than the ISI threshold. Network bursts, or a “burst of bursts,” were detected using the same algorithm but on burst events instead of spikes. A *post-hoc* criterion of a minimum number of activated channels per network burst was specified as 20% of the recording channels. This corresponds to 25 channels in our array, but a value of 10 channels was used to improve consistency with the ISI_*N* = 10_-threshold detector.

#### Rank surprise detector

The non-parametric *Rank Surprise* algorithm was acquired from Matlab code provided with the original publication by Gourevitch and Eggermont (2007). As data in the original paper were recorded in the cortex of an anesthetized cat *in vivo*, two default parameters were changed to improve consistency with the other detectors: The minimum number of spikes and maximum ISI within a burst were changed to 5 spikes and 100 ms, respectively. However, the default values of 3 spikes and the 75th percentile of ISI produced similar results. A method to identify network bursts was not provided. Therefore, we identified a network burst simply whenever at least 10 single channel bursts overlapped temporally.

## Results

Bursts were identified in 1-h recordings of spontaneous activity from primary cortical cultures using the ISI_*N*_-threshold detector with *N* = 10 (see Methods). Figure 1 shows typical neuronal network activity. Bursts showed a variety of sizes and inter-burst-intervals. Tonic activity commonly occurred in a subset of channels to varying degrees. Some channels showed elevated tonic activity throughout the duration of a recording, while others showed intermittent tonic activity. Recording channels were ordered by the overall firing rate in order to better observe the occurrence of bursts, indicated by the columns of spikes in the raster plot.

The method to find the ISI_*N* = 10_ threshold used to detect bursts in Figure 1 is depicted in Figure 2. The deflection in the probability curve suggests the existence of more than one type of activity state, such as bursting (low ISI_*N*_) and non-bursting (high ISI_*N*_; contributions from tonic activity) regimes. Increasing *N* increased the amount of deflection in the curve (Figures 2A, 4). This will add precision when determining a threshold and improve performance at the expense of increasing the criterion for the minimum number of spikes per burst. The ISI_*N* = 10_ threshold was 50 ms for the represented data and 150 ± 100 ms (mean ± *SD*) across 9 cultures. For comparison, an ISI_*N* = 2_ threshold was approximately 20 ms (Figure 2A, large black arrow) and 35 ± 45 ms (mean ± *SD*) across 9 cultures. The probability distributions and thresholds will depend on the overall number of neurons and their firing profiles, which may vary in time. The additional spikes that would be classified as composing a burst for ISI_*N* = 2_ but not for ISI_*N* = 10_ are presented as cyan circles in Figures 2B,C. While some of these spikes appear to be false positives, the close alignment of others may represent bursts, or perhaps assemblies, with fewer than 10 spikes.

**Figure 3:**
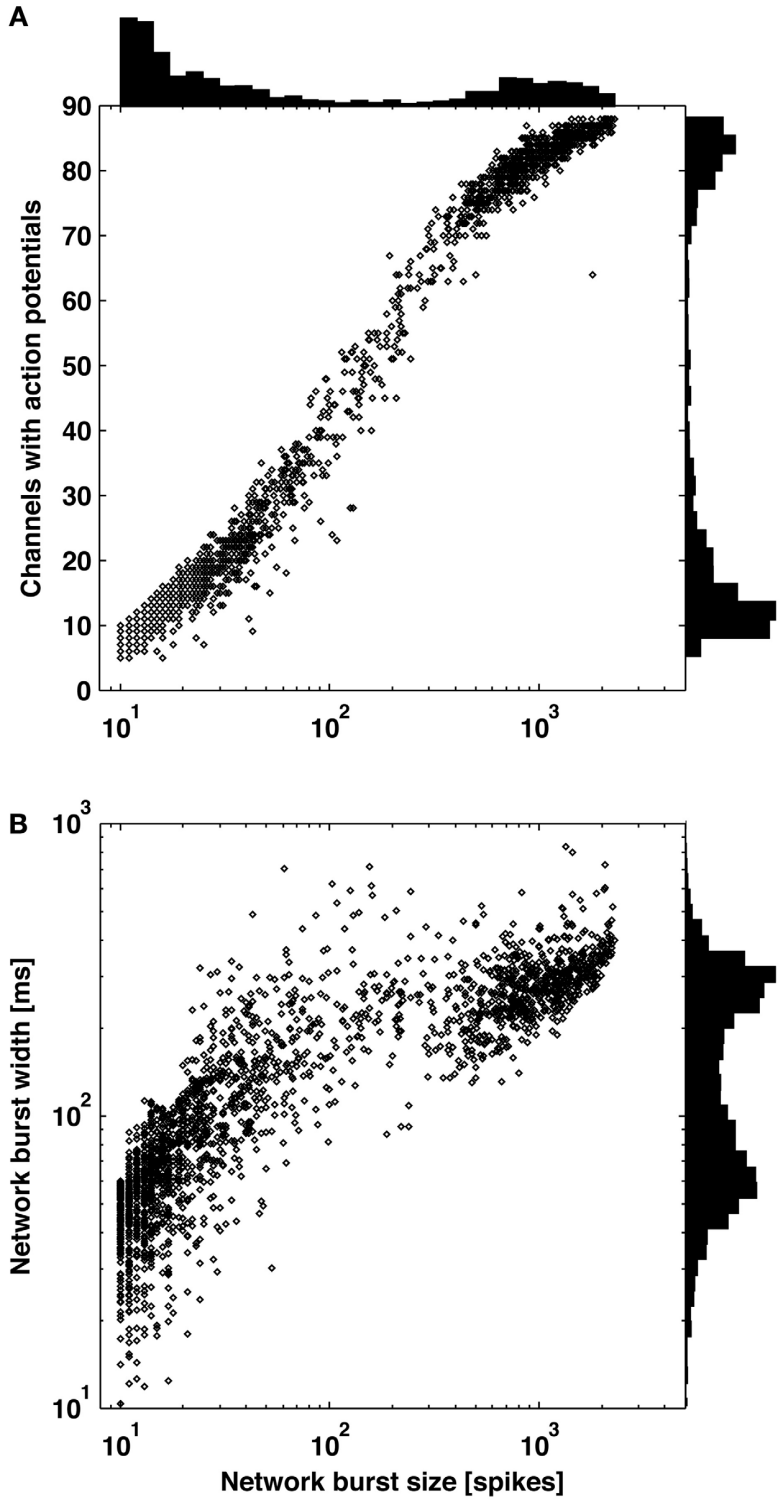
**Distribution of burst sizes**. Increasing burst size correlated to increasing number of contributing channels **(A)** and increasing burst width **(B)**. Larger bursts covering the majority of channels and smaller bursts covering a subset of channels are clearly distinguishable by observing valleys in each of the three histograms.

In 6 out of 9 cultures, a clear distinction between large bursts covering the majority of channels and smaller bursts covering a subset of channels was apparent from observing distributions of burst size (number of spikes or elapsed time) or the number of contributing channels (Figure 3). Small bursts averaged less than 1 spike per recording channel, indicating they would be difficult to identify using existing detectors. A continuum of smaller bursts existed without a clear cutoff between bursting and non-bursting regimes. This suggests using caution when making assumptions about burst dimensions or “surprise” criteria for burst detection. Considering the small bursts represented 80 percent of all bursts and 10 percent of the spiking for this culture, much diversity in network information processing may be overlooked if only large bursts are detected and analyzed. Small bursts in the other 5 cultures represented 76, 45, 26, 25, and 12% of all bursts for each culture. From an information theory perspective, the extreme cases of no neuron or all neurons firing (i.e., the largest bursts) provide no information content, or only 1 bit if combined as on and off states. Smaller bursts then theoretically hold more information content. A previous experiment demonstrates the utility of small bursts, where electrically evoked bursts were used to instruct motor output in an embodied cultured network (Bakkum et al., 2008). Successful goal-directed behavior, based on plasticity in the spatio-temporal burst structure, was possible only when using a stimulation electrode that evoked small bursts.

**Figure 4:**
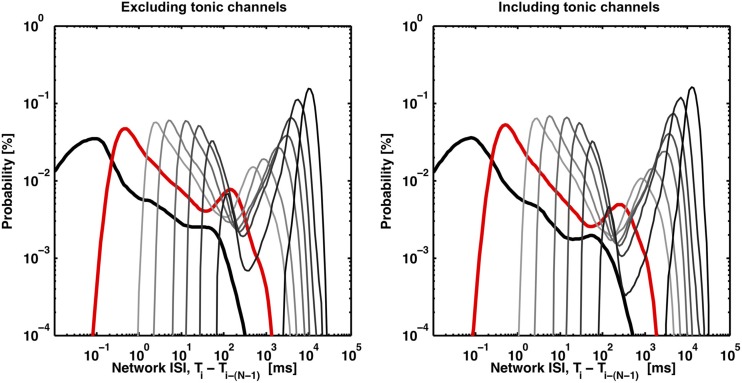
**Valleys in the bimodal ISI_*N*_ probability distributions deepen with increasing *N* (light gray to dark gray) or with the inclusion of channels exhibiting tonic spiking (right panel)**. The distributions (black, red, light gray to dark gray) correspond to *N* equal to 2, 10, 50, 100, 250, 500, 1000, 2000, and 4000. The first peak disappears as *N*, the minimum burst size threshold, approaches the maximum burst size (~4000 spikes), which represents the trivial case of no spikes being within a burst. 8 out of 102 channels were identified as tonic according to the method presented in Figure 5. The inclusion of tonic channels can improve valley identification with a tradeoff of increased risk of identifying consecutive bursts as a single burst.

**Figure 5:**
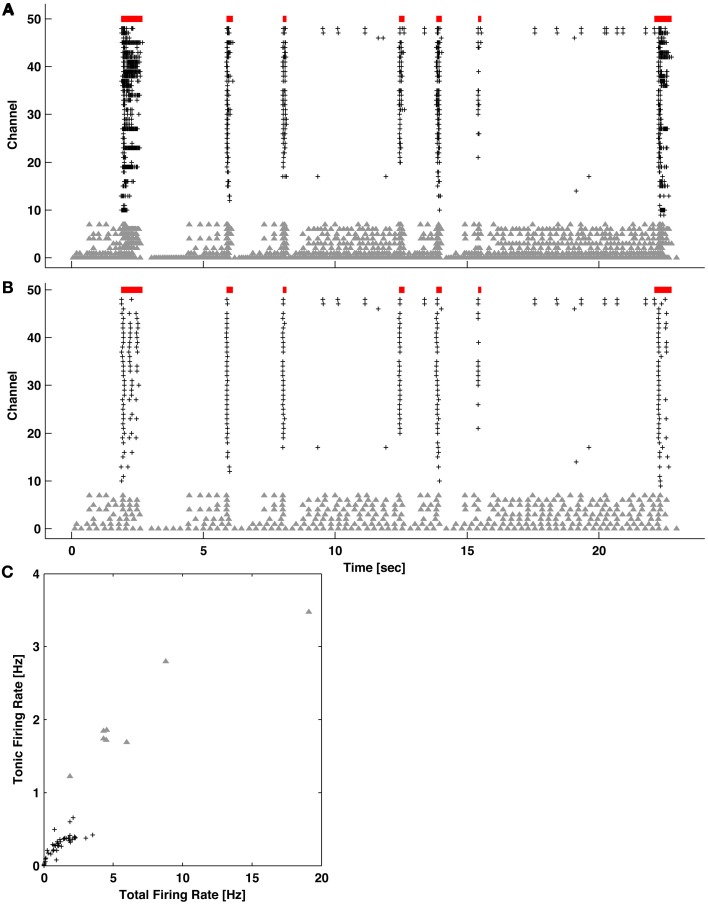
**Quantifying levels of tonic activity. (A)** Example network activity exhibiting bursting (red bars) and channels with high levels of tonic spiking (gray triangles). Spikes on all other active channels are indicated by black pluses. **(B)** The same network activity after introducing an artificial refractory period of 250 ms. Spikes during bursting became preferentially removed. **(C)** For each channel, the tonic (from **B**) vs. total (from **A**) firing rate is plotted. Channels with elevated tonic firing (gray triangles) match visual observations in **(A)** and **(B)**.

While the ISI_*N*_-threshold detector was robust in the presence of tonic activity, which in fact helped to form the second peak and valley in the logarithmic ISI_*N*_ histograms that were used to determine the ISI_*N*_ threshold (Figure 4), strong tonic firing on too many channels may compromise detection performance. For example, sustained tonic spiking could cause neighboring bursts to be falsely identified as a single burst. Also, tonic spiking on multiple channels could produce false-positive bursts. In any case, channels exhibiting high tonic activity are readily apparent from a visual inspection of raster plots (Figure 1). As an optional pre-processing step, these channels can be excluded. To make exclusion less subjective and more automated, a method to quantify the amount of tonic activity on a given recording channel is depicted in Figure 5. Here, a “tonic firing rate” was calculated by enforcing an arbitrarily chosen refractory period of 250 ms, whereby any spike following within the refractory period was excluded. This filtered out most spikes occurring during bursts while preserving much of the tonic spiking occurring between bursts. Channels with high tonic firing rates corresponded well to visual observations (Figure 5). A suitable choice of refractory period is longer than the average ISI of a neuron and shorter than the average inter-burst-interval.

## Discussion

### Comparison to existing detectors

**Figure 6:**
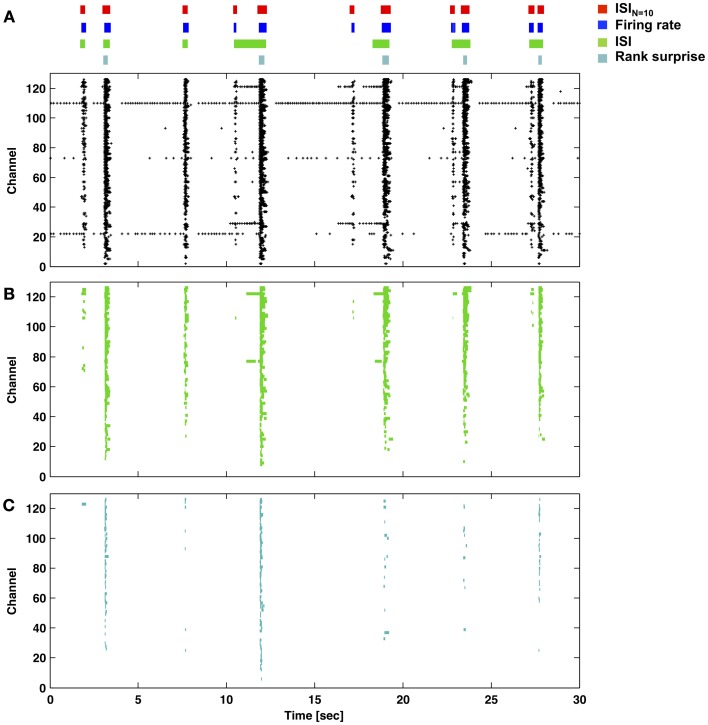
**Performance of the ISI_*N* = 10_-threshold detector compared to example rate-threshold, ISI-threshold, and Rank Surprise detectors. (A)** Burst times and durations for each detector (color bars and legend) applied to the network activity presented in the raster plot (each marker is one spike). The ISI_*N* = 10_-threshold and rate-threshold detectors were performed directly on the network spike train. The ISI-threshold and Rank Surprise detectors were performed on single-channel spike trains in a first stage. The respective single-channel burst events in **(B)** and **(C)** were then combined in a second stage to identify network bursts as described in the Methods.

**Figure 7:**
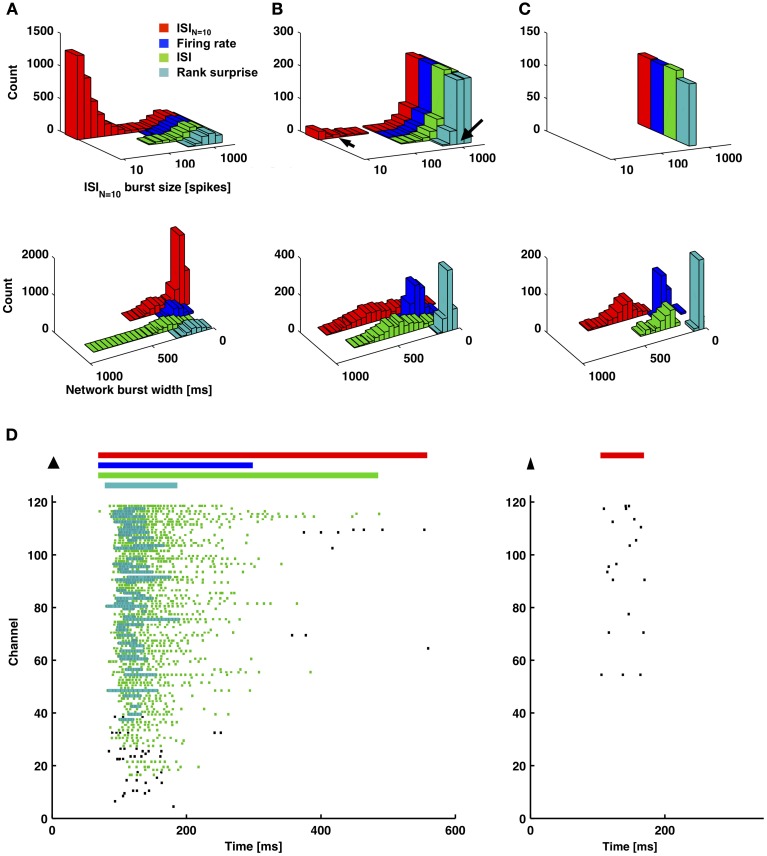
**An ISI_*N* = 10_-threshold detector identified small-sized bursts, and measured burst durations were shorter for a firing-rate-threshold or a rank surprise detector. (A–C)** Each method was applied to 1-h long recordings from 3 cultures that each had different amounts of small bursts. The percentage of small bursts out of the total number of bursts, according to the ISI_*N* = 10_-threshold detector, were 80, 12, and 0% for **(A–C)**, respectively. Each detector identified the largest bursts, and the ISI_*N* = 10_-threshold detector identified smaller bursts (left column). Since different detectors assign different durations to the same burst, one detector (ISI_*N* = 10_) was chosen as a reference (x-axis). In this manner, the same burst will be plotted at the same x-location, and its occurrence can be compared across detectors. Detected bursts had shorter durations for the firing-rate-threshold and rank surprise detectors. This was especially noticeable for cases with fewer short-duration small bursts in **(B)** and **(C)** (right column and **D**). Arrows in **(B)** correspond to examples of a large and a small burst in **(D)**. **(D)** Plotted in **(D)** are durations of identified network bursts using each method (colored bars; top) and identified first-stage single-channel bursts using the ISI and Rank surprise methods (colored raster plots; each dot is a spike).

**Figure 8:**
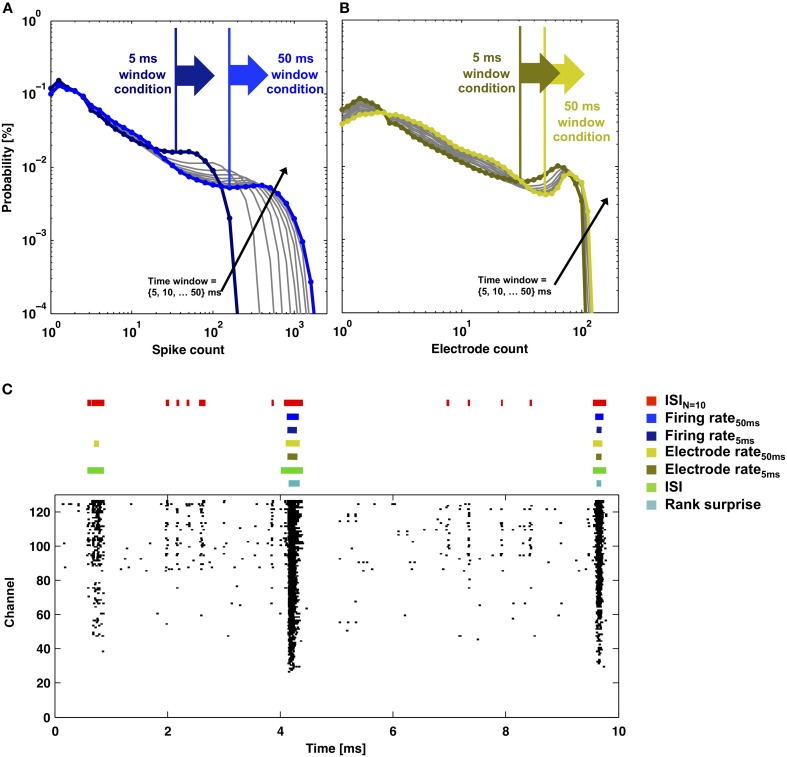
**Rate-thresholds and burst detection for data presented in Figure 2**. The probability distributions of **(A)** the total number of spikes or **(B)** the total number of electrodes that detected a spike within a time window between 5 and 50 ms in duration (lines) are plotted. Elevated firing during network bursting corresponds to higher spike or electrode counts, and the large arrows indicate the rate-threshold for burst detection used in **(C)**. **(C)** Detector performance for a segment of network activity (black dots). Colored bars indicate detected bursts for each burst detector.

Figures 6, 7 provide a comparison of the ISI_*N*_-threshold detector with detectors representative of the rate-threshold, ISI-threshold, and surprise methods by Ji and Wilson, Pasquale et al., and Gourevitch and Eggermont, respectively (Mukai et al., 2003; Gourevitch and Eggermont, 2007; Ji and Wilson, 2007; Pasquale et al., 2010). While the rate-threshold and ISI_*N*_-threshold methods operated directly on the cumulative network spike train, the ISI-threshold and the Rank Surprise methods required a first-stage single-channel burst detection step prior to identification of a network burst (Figures 6B,C). The ISI_*N* = 10_ threshold was equal to 140 ms in Figure 6 and 50, 250, and 350 ms in Figures 7A–C, respectively. The rate threshold for a 50 ms time window was equal to 8 spikes in Figure 6 and 160, 80, and 10 spikes in Figures 7A–C, respectively. Rate-threshold detector performance was similar for a 5 ms time window (see Figure 8). Refer to the Methods for implementation details. To compare performance in different culture preparations, Figure 7 contains data from 3 cultures whose small bursts were, according to the ISI_*N* = 10_-threshold detector, 80, 12, or 0% of the total number of bursts per each culture. Data from the culture in Figure 7A is also presented in Figures 1–3.

All methods detected the largest network bursts, and the ISI_*N*_-threshold detector also detected smaller network bursts (Figures 7A–C; left column). In Figure 7A, 20% of the bursts detected by the ISI_*N* = 10_-threshold method were considered to be large bursts (c.f. Figure 3). This is consistent with the ISI-threshold, rate-threshold, and Rank Surprise methods detecting 18, 16, and 11% of the total bursts identified by the ISI_*N* = 10_-threshold method, respectively. For Figure 7B, the values were: 88% large bursts; 90, 90, and 76% of the total detected. For Figure 7C, the values were: 100% large bursts; 100, 100, and 93% of the total detected. The ISI-threshold detector struggled with tonic activity, whereby some bursts had artificially extended durations that sometimes merged neighbored bursts (Figure 6). The authors of the ISI-threshold detector acknowledged this situation (Pasquale et al., 2010), and they and others provided a *post-hoc* solution described below. Merged bursts are also possible with the ISI_*N*_-threshold detector, especially when the ISI_*N*_ threshold is relatively large. The rate-threshold detector performed well. However, burst durations were consistently underestimated (Figures 7A–C; right column), and the smallest bursts in a more challenging dataset were not detected (Figure 8, Supplemental Figure 1). Furthermore, the inability to accurately assign burst boundaries can be a drawback for some applications. Interestingly, rate-threshold detection with a shorter 5 ms time window underestimated burst duration to a greater extent (Figure 8). A rate-threshold detector based on the number of electrodes or the number of (spike-sorted) neurons displaying activity, as opposed to the number of spikes recorded, produced similar results (Figure 8, Supplemental Figure 1). Rate-threshold detector performance will improve with increasing numbers of recording channels: time windows that contain bursts will summate to multiplicatively larger values than windows containing lower frequency tonic activity.

The Rank Surprise method detected the fewest bursts and most underestimated burst duration (Figure 7). A commonly touted advantage of surprise methods over other methods is their ability to assign significance values to each burst. This is indeed a desirable feature if detection performs adequately. However, underestimating the number and duration of bursts appears to be inherent to the algorithm: The underlying Poisson or rank distribution, or what is “unsurprising,” is created including the “surprising” spikes that are in bursts. Therefore, spikes in the tails of bursts, where ISIs are longer than in the burst core, will no longer be “surprising.” In Figures 6, 7D, the tails of bursts are clearly excluded. This effect will be amplified for preparations with large percentages of spikes occurring within bursts, such as *in vitro* networks (90 ± 12% of spikes were within network bursts according to the ISI_*N* = 10_-threshold detector; mean ± *SD*, 9 cultures). Lowering the default threshold for what would be surprising entailed the detection of more spikes in bursts. However, the smallest bursts were still not detected, and, at the same time, wide stretches of tonic activity were now erroneously identified as bursts. Perhaps improved performance could be achieved by considering more than one distribution, with one representing a bursting regime, and then measure “surprise” with respect to modeled ISI distributions of the non-bursting regimes. The durations of the bursts detected with the Rank Surprise and rate-threshold detectors were 35 ± 15% and 49 ± 15%, respectively, of the durations of the same bursts identified by the ISI_*N* = 10_-threshold detector (mean ± *SD* for 9 cultures).

In their most basic form, rate-threshold and ISI_*N*_-threshold (including ISI-threshold) methods offer two sides of the same coin. The former measures the number of spikes detected within a fixed window of time while the latter measures how much time elapsed for a fixed number of spikes to occur. To emphasize the point, the rate-threshold detector in Figure 6 is related to an ISI_*N*_-threshold detector having an ISI_*N* = 8_ threshold equal to 50 ms. The choice of number of spikes or time window as a threshold is arbitrary, but the former creates artificial time points for burst boundaries at threshold crossings, while the latter allows the assignment of specific time points for the first and last spike in putative bursts.

The “discharge density” histograms (i.e., probability distributions of firing rates) (Kaneoke and Vitek, 1996; Ji and Wilson, 2007) provide a means to flip the coin and automatically determine both thresholds. By using a fixed firing-rate histogram time window, the threshold *N* (number of spikes) was selected at the valley separating high and low firing rate peaks in the discharge density plot (c.f. Figure 8). Flipping the coin, the method presented here can use the *N* found above to select an ISI_*N*_ threshold (c.f. Figure 2). In turn, the ISI_*N*_ threshold can update the time window of a new discharge density histogram in order to update *N*, thus continuing an iterative process. For our data, *N* and the ISI_*N*_ threshold converged, but whether or not these values are optimal for burst detection is debatable. For example for data presented in Figures 1 and 6, the values converged to an ISI_*N* = 260_ threshold equal to 200 ms and an ISI_*N* = 17_ threshold equal to 220 ms, respectively. Detection appeared to work well. However, the small bursts in Figure 1 (i.e., less than *N* = 260) could no longer be detected, and longer ISI_*N*_ thresholds led to some merged bursts. For data in Figure 1, the process appeared to find the border between large and small bursts (c.f. compare *N* = 260 to Figure 3). Alternatively, the simultaneous use of multiple pairs of *N* and ISI_*N*_ thresholds is expected to improve detection performance. A simple example is given in the next paragraph, where an additional ISI_*N* = 2_ threshold helped to reduce the number of merged bursts. As a last note, the valleys in the probability distribution histograms provided an intuitive choice for a border but are not necessarily optimal. Finding optimal borders, and similarly precise measures of performance, requires having a ground truth about when bursts occur, which, unfortunately, does not exist.

Tonic activity can merge neighboring bursts for ISI_*N*_- and ISI-threshold detectors. Excluding tonic channels in a pre-processing stage, as discussed in the Results, offers one solution. Alternatively, merged bursts could be separated in a post-processing stage by finding time points of lowest firing between putative bursts as described by (Wagenaar et al., 2005; Pasquale et al., 2010); this technique is inherently done by rate-threshold detectors. However, whether or not putative bursts are distinct or different phases of the same burst was not always apparent. A further alternative that avoids pre- or post-processing steps is to introduce a second parameter during burst detection. For example, an ISI_*N* = 2_ threshold of 20 ms (50 Hz) effectively broke into pieces the tonic spike trains, which did not sustain 50 Hz firing rates. Since an ISI_*N* = 2_ threshold may be difficult to identify (c.f. Figure 2A), such a threshold may need to be adjusted in an *ad-hoc* manner for different preparations.

## Conclusion

A network burst was defined as a period of elevated firing within or across multiple channels. Creating a network burst detector then consists of finding appropriate thresholds to separate periods of low and high firing states. We proposed an ISI_*N*_-threshold burst detector, where ISI_*N*_ is the inter-spike-interval between every *N*th spike in the network. The ISI_*N*_-threshold burst detector performed well with our data, but we acknowledge that other detectors may be better suited for other types of data. For example, the ISI_*N*_-threshold burst detector struggled most when multiple channels exhibited intermittent and irregular activity. For single neuron bursts, an ISI-threshold burst detector (ISI_*N* = 2_) sufficiently distinguishes bursting regimes (Pasquale et al., 2010).

Our approach offered a number of useful features including: a simple and computationally efficient implementation, no need for *ad-hoc* or *post-hoc* criteria, and precise assignment of burst boundary time points. The choice of *N* is not obvious, however, and could be considered to be an *ad-hoc* decision based on familiarity with the structure of the spike trains. We chose *N* = 10 as it was large enough to demonstrate a deepening of valleys in the probability distributions (Figure 2A) and small enough to allow a wide range of burst sizes to be detected. Choosing *N* equal to the minimum burst size would give the least sensitivity to tonic or noisy channels, but a minimum burst size may not be obvious (c.f. Figure 3). On the other hand, choosing the smallest *N* that provides easily separable peaks in the ISI_*N*_ probability distribution would not bias detection toward larger bursts, allowing identification and analysis of a greater range of neuronal and network dynamics.

### Conflict of interest statement

The authors declare that the research was conducted in the absence of any commercial or financial relationships that could be construed as a potential conflict of interest.

## Supplementary material

Supplemental Figure 1 **Rate-thresholds and burst detection for spike-sorted data presented in Figures 2 and 8**. The probability distributions of **(A)** the total number of neurons spiking or **(B)** the total number of electrodes that detected a spike within a time window between 5 and 50 ms in duration (lines) are plotted. Elevated firing during network bursting corresponds to higher neuron or electrode counts, and the large arrows indicate the rate-threshold for burst detection used in **(C)** and **(D)**. Hundred and two electrodes detected spikes, and from these, 62 individual neurons were manually identified (spike-sorted) based on having distinct spatio-temporal activity profiles (Franke et al., 2012a). **(C)** Histograms of neuron or electrode counts for 5 or 50 ms time windows for the network activity presented in **(D)**. The rate-thresholds detected in **(A)** and **(B)** are plotted as dotted lines, and a burst is detected whenever a count exceeds the rate-threshold. **(D)** Detector performance for a segment of network activity (black dots). Colored bars indicate detected bursts for each burst detector.

Supplementary Code I **Matlab code for creating ISI_*N*_ histogram plots in order to choose an ISI_*N*_ threshold**.

Supplementary Code II **Matlab code for ISI_*N*_ burst detection**.
