# Supplementary material for: Parameters for burst detection
Source: Front Comput Neurosci. 2014 Jan 13;7:193. doi: 10.3389/fncom.2013.00193 (PMC3915237; doi:10.3389/fncom.2013.00193)
Supplement: Supplemental Figure 1 — Rate-thresholds and burst detection for spike-sorted data presented in Figures 2 and 8. The probability distributions of (A) the total number of neurons spiking or (B) the total number of electrodes that detected a spike within a time window between 5 and 50 ms in duration (lines) are plotted. Elevated firing during network bursting corresponds to higher neuron or electrode counts, and the large arrows indicate the rate-threshold for burst detection used in (C) and (D). Hundred and two electrodes detected spikes, and from these, 62 individual neurons were manually identified (spike-sorted) based on having distinct spatio-temporal activity profiles (Franke et al., 2012a). (C) Histograms of neuron or electrode counts for 5 or 50 ms time windows for the network activity presented in (D). The rate-thresholds detected in (A) and (B) are plotted as dotted lines, and a burst is detected whenever a count exceeds the rate-threshold. (D) Detector performance for a segment of network activity (black dots). Colored bars indicate detected bursts for each burst detector. [file Presentation1.PDF]

Parameters for burst detection

Supplementary Material

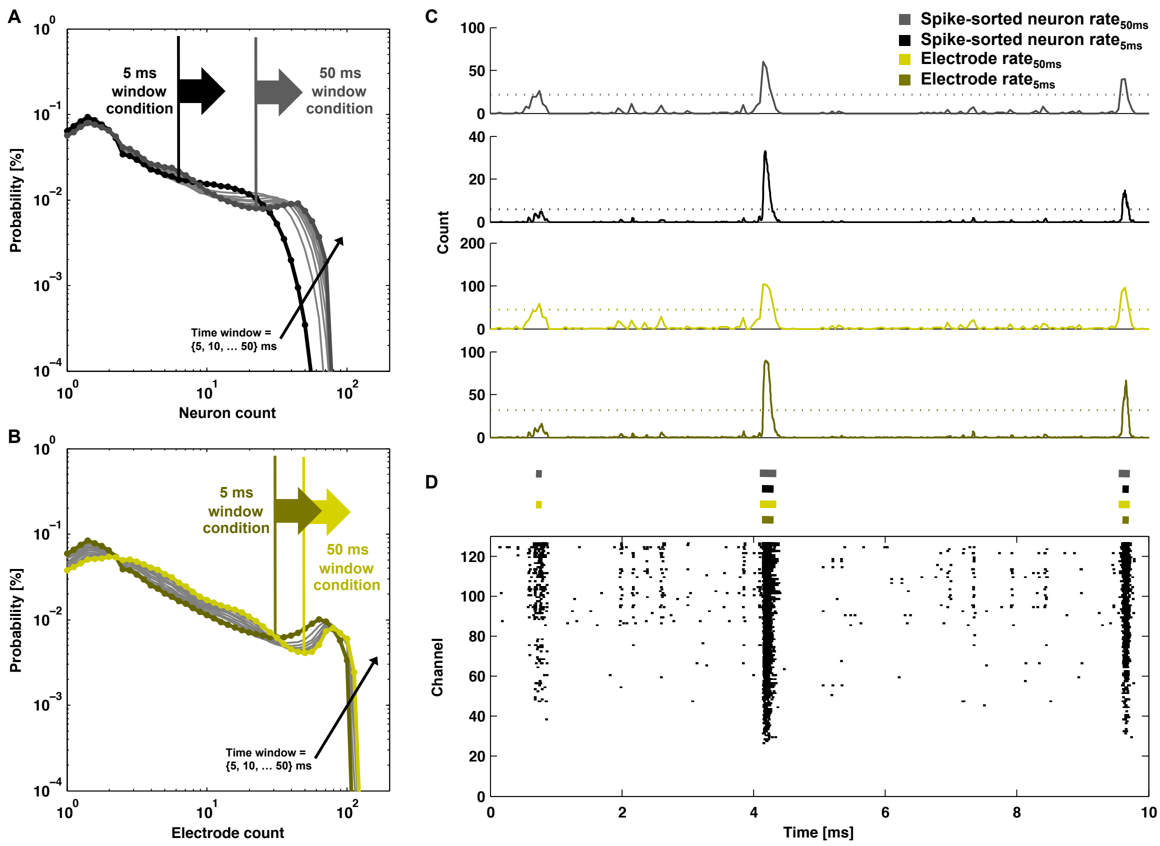

Supplemental Figure 1 | Rate-thresholds and burst detection for spike-sorted data presented in Figures 2 and 8. The probability distributions of **(A)** the total number of neurons spiking or **(B)** the total number of electrodes that detected a spike within a time window between 5 and 50 ms in duration (lines) are plotted. Elevated firing during network bursting corresponds to higher neuron or electrode counts, and the large arrows indicate the rate-threshold for burst detection used in **(C)** and **(D)**. Hundred and two electrodes detected spikes, and from these, 62 individual neurons were manually identified (spike-sorted) based on having distinct spatio-temporal activity profiles (Franke et al., 2012a). **(C)** Histograms of neuron or electrode counts for 5 or 50 ms time windows for the network activity presented in **(D)**. The rate-thresholds detected in **(A)** and **(B)** are plotted as dotted lines, and a burst is detected whenever a count exceeds the rate-threshold. **(D)** Detector performance for a segment of network activity (black dots). Colored bars indicate detected bursts for each burst detector.

Supplementary Code I | Matlab code for creating ISI_N histogram plots in order to choose an ISI_N threshold.

```
function HistogramISIn( SpikeTimes, N, Steps )
% ISI_N histogram plots
% © Douglas Bakkum, 2013
%
%
%   HistogramISIn( SpikeTimes, N, Steps )
%       'SpikeTimes' [sec]     % Vector of spike times.
%       'N'                    % Vector of values for plotting ISI_N histograms.
%       'Steps'  [sec]         % Vector of histogram edges.
%
%   Steps should be of uniform width on a log scale. Note that histograms
%   are smoothed using smooth.m with the default span and lowess method.
%
%
%   Example code:
%       SpikeTimes          =  ---- ;               % Load spike times here.
%       N                   = [2:10];               % Range of N for ISI_N histograms.
%       Steps               = 10.^[-5:.05:1.5]; % Create uniform steps for log plot.
%       HistogramISIn(SpikeTimes,N,Steps)     % Run function
%

figure; hold on
map  = hsv(length(N));

cnt  = 0;
for FRnum = N
    cnt     = cnt + 1;
    ISI_N = SpikeTimes( FRnum:end ) - SpikeTimes( 1:end-(FRnum-1) );
    n       = histc( ISI_N*1000, Steps*1000 );
    n       = smooth( n, 'lowess' );
    plot( Steps*1000, n/sum(n), '.-', 'color', map(cnt,:) )
end

xlabel 'ISI, T_i - T_{i-(N-1)} _{ }} [ms]'
ylabel 'Probability [%]'
set(gca,'xscale','log')
set(gca,'yscale','log')
```

Supplementary Code II | Matlab code for ISI_N burst detection.

```
function [Burst SpikeBurstNumber] = BurstDetectISIn( Spike, N, ISI_N )
% ISI_N burst detector
% © Douglas Bakkum, 2013
%
%   [Burst SpikeBurstNumber] = burstDetectISIn( Spike, N, ISI_N)
%
%      'Spike' is a structure with members:
%           Spike.T                         Vector of spike times [sec]
%           Spike.C (optional)              Vector of spike channels
%
%      'N' spikes within 'ISI_N' [seconds] satisfies the burst criteria.
%
%
%      Returns Burst information and the Burst Number for each spike time:
%
%           Burst.T_start                   Burst start time [sec]
%           Burst.T_end                     Burst end time   [sec]
%           Burst.S                         Burst size (number of spikes)
%           Burst.C                         Burst size (number of channels)
%
%           SpikeBurstNumber                Burst number for each Spike;
%                                           '-1' if a spike is not in a burst.
%
%
%   Example code:
%
%       Spike.T     = ---- ;                % Load spike times here.
%       Spike.C     = ---- ;                % Load spike channels here.
%       N           =    10;                % Set N
%       ISI_N       =  0.10;                % Set ISI_N threshold [sec]
%                                           % Run the detector
%       [Burst Spike.N] = BurstDetectISIn( Spike, N, ISI_N );
%
%       % Plot results
%       figure,  hold on
%
%       % Order y-axis channels by firing rates
%       tmp = zeros( 1, max(Spike.C)-min(Spike.C) );
%       for c = min(Spike.C):max(Spike.C)
%         tmp(c-min(Spike.C)+1) = length( find(Spike.C==c) );
%       end
%       [tmp ID] = sort(tmp);
%       OrderedChannels = zeros( 1, max(Spike.C)-min(Spike.C) );
%       for c = min(Spike.C):max(Spike.C)
%         OrderedChannels(c-min(Spike.C)+1) = find( ID==c-min(Spike.C)+1 );
%       end
```

```
%
%       % Raster plot
%       plot( Spike.T, OrderedChannels(1+Spike.C), 'k.' )
%       % set( gca, 'ytick', (min(Spike.C):max(Spike.C))+1, 'yticklabel', ...
%       %              ID-min(ID)+min(Spike.C) ) % set yaxis to channel ID
%
%       % Plot times when bursts were detected
%       ID          = find(Burst.T_end<max(Spike.T));
%       Detected    = [];
%       for i=ID
%         Detected = [ Detected  Burst.T_start(i)  Burst.T_end(i)  NaN ];
%       end
%       plot( Detected, 128*ones(size(Detected)), 'r', 'linewidth', 4 )
%
%       xlabel 'Time [sec]'
%       ylabel 'Channel'
%

fprintf('Beginning burst detection.\n');

% %% Find when the ISI_N burst condition is met

   % Look both directions from each spike
   dT = zeros(N,length(Spike.T))+inf;
   for j = 0:N-1
          dT(j+1,N:length(Spike.T)-(N-1)) = Spike.T( (N:end-(N-1))+j ) - ...
                                          Spike.T( (1:end-(N-1)*2)+j );
   end
   Criteria = zeros(size(Spike.T));    % Initialize to zero
   Criteria(  min(dT)<=ISI_N ) = 1;    % Spike passes condition if it is
                                       % included in a set of N spikes
                                       % with ISI_N <= threshold.

% %% Assign burst numbers to each spike

   SpikeBurstNumber       = zeros(size(Spike.T)) - 1;   % Initialize to '-1'
   INBURST                =  0;                % In a burst (1) or not (0)
   NUM_                   =  0;                % Burst Number iterator
   NUMBER                 = -1;                % Burst Number assigned
   BL                     =  0;                % Burst Length

   for i = N:length(Spike.T)

          if INBURST == 0                      % Was not in burst.
              if  Criteria(i)                  % Criteria met, now in new burst.
                   INBURST  = 1;               % Update.
                   NUM_     = NUM_ + 1;
                   NUMBER   = NUM_;
                   BL       = 1;
              else                             % Still not in burst, continue.
```

```
                % continue %
            end

        else                                % Was in burst.
            if ~ Criteria(i)                % Criteria no longer met.
                INBURST   =  0;             % Update.
                if BL<N                     % Erase if not big enough.
                    SpikeBurstNumber(SpikeBurstNumber==NUMBER) = -1;
                    NUM_   = NUM_ - 1;
                end
                NUMBER   = -1;

            elseif diff(Spike.T([i-(N-1) i])) > ISI_N  &&  BL >= N
                % This conditional statement is necessary to split apart
                % consecutive bursts that are not interspersed by a tonic spike
                % (i.e. Criteria == 0). Occasionally in this case, the second
                % burst has fewer than 'N' spikes and is therefore deleted in
                % the above conditional statement (i.e. 'if BL<N').
                %
                % Skip this if at the start of a new burst (i.e. 'BL>=N'
                % requirement).
                %
                NUM_     = NUM_ + 1;        % New burst, update number.
                NUMBER   = NUM_;
                BL       = 1;               % Reset burst length.

            else                            % Criteria still met.
                BL       = BL + 1;          % Update burst length.

            end
        end

        SpikeBurstNumber(i)  = NUMBER;      % Assign a burst number to
                                            % each spike.
end

% %% Assign Burst information

fprintf('Assigning Burst information.\n');

MaxBurstNumber = max(SpikeBurstNumber);

Burst.T_start     = zeros(1,MaxBurstNumber); % Burst start time [sec]
Burst.T_end       = zeros(1,MaxBurstNumber); % Burst end time   [sec]
Burst.S           = zeros(1,MaxBurstNumber); % Size (total spikes)
Burst.C           = zeros(1,MaxBurstNumber); % Size (total channels)

for i = 1:MaxBurstNumber
        ID                  = find( SpikeBurstNumber==i );
        Burst.T_start(i)    = Spike.T(ID(1));
```

```
            Burst.T_end(i)      = Spike.T(ID(end));
            Burst.S(i)          = length(ID);
            if isfield( Spike, 'C' )
                Burst.C(i)      = length( unique(Spike.C(ID)) );
            end
end

fprintf('Finished burst detection using %0.2f minutes of spike data.\n', ...
        diff(Spike.T([1 end]))/60);
```
